# Closing the defect after gastric endoscopic full‐thickness resection with a novel linear-traction metal clip

**DOI:** 10.1055/a-2740-3607

**Published:** 2025-11-28

**Authors:** Qianqian Wang, Wenlei Li, Yuxuan Chen, Jianfen Wu, Shuo Zhang

**Affiliations:** 1 587400 Department of Gastroenterology, The Second Affiliated Hospital of Zhejiang Chinese Medical University, Hangzhou City, China; 2 Key Laboratory of Traditional Chinese Medicine for the Treatment of Intestine-Liver of Zhejiang Province, Hangzhou City, China

A 59-year-old male presented to our hospital due to a mucosal elevation in the lower body of the stomach ( Fig. 1 ). EUS revealed the tumor extending to the serosal layer. To achieve complete resection, we performed a full-thickness resection. Given the large size of the lesion, prolonged operative time, and proximity to the antrum with significant peristaltic movement, the wound circumference was long, with marked mucosal oedema and inflammatory reaction. The overall structure was invaginated, resulting in incomplete endoscopic exposure and difficulty with closure ( Fig. 2 , Video 1 ). We initially used a three-arm clip to partially close the wound, then fixed the thread at the edge of the wound using metal clips to the normal mucosa, and performed pulling in vitro to achieve side-to-side apposition, providing a clearer operative view ( Video 1 ). After completing the wound closure with metal clips, we removed the clips used for fixing the thread ( Fig. 3 ). The procedure was smooth, and the patient experienced no adverse events. The novel linear-traction metal clip closure device is convenient, economical, safe, and effective. It helps achieve the closure of difficult wounds and provides valuable application references for similar surgeries.

**Fig. 1:**
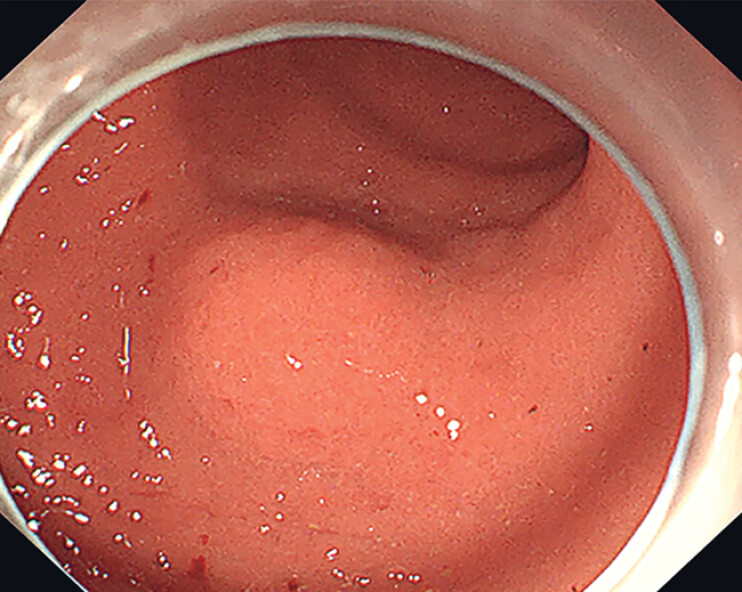
A mucosal elevation in the lower body of the stomach.

**Fig. 2:**
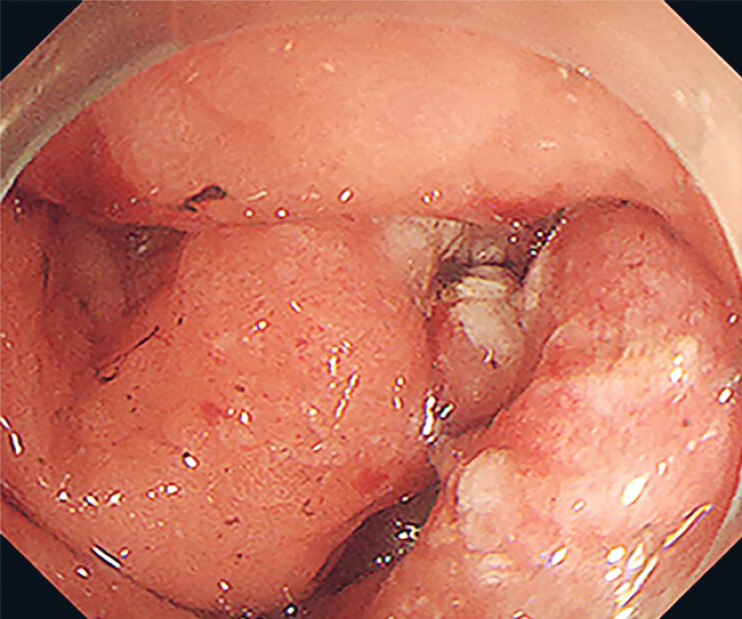
Long surgical incision with significant mucosal oedema and inflammation.

**Fig. 3:**
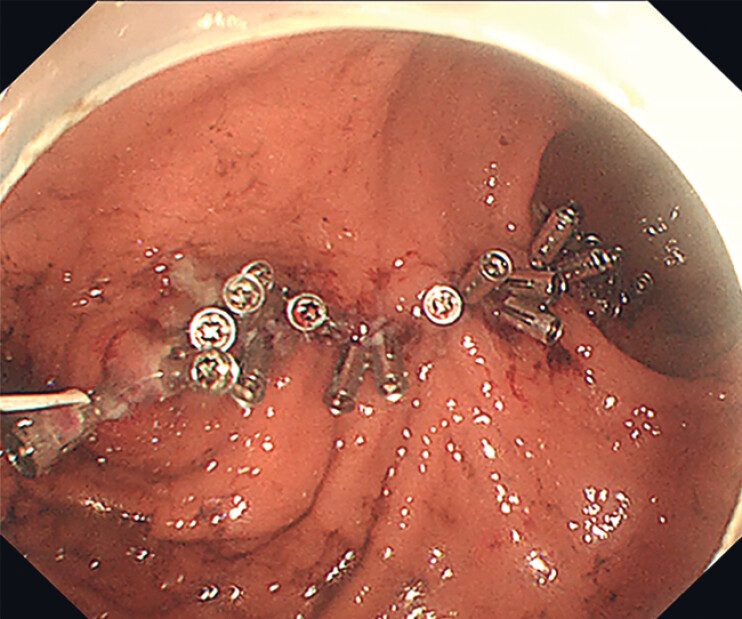
Complete closure of the difficult defect was achieved.

Using the linear-traction metal clip to close the defect after gastric endoscopic full-thickness resection.Video 1

Endoscopy_UCTN_Code_CPL_1AH_2AZ_3AF

## Notes

Endoscopy E-Videos https://eref.thieme.de/e-videos
*E-Videos* is an open access online section of the journal *Endoscopy* , reporting on interesting cases and new techniques in gastroenterological endoscopy. All papers include a high-quality video and are published with a Creative Commons CC-BY license. Endoscopy E-Videos qualify for HINARI discounts and waivers and eligibility is automatically checked during the submission process. We grant 100% waivers to articles whose corresponding authors are based in Group A countries and 50% waivers to those who are based in Group B countries as classified by Research4Life (see: https://www.research4life.org/access/eligibility/ ). This section has its own submission website at https://mc.manuscriptcentral.com/e-videos .

